# Long-term follow-up in adults after tetralogy of Fallot repair

**DOI:** 10.1186/s12947-018-0146-7

**Published:** 2018-10-29

**Authors:** Natalia Dłużniewska, Piotr Podolec, Maciej Skubera, Monika Smaś-Suska, Jacek Pająk, Małgorzata Urbańczyk-Zawadzka, Wojciech Płazak, Maria Olszowska, Lidia Tomkiewicz-Pająk

**Affiliations:** 1 Department of Cardiac and Vascular Disease, Collegium Medicum, Jagiellonian University, John Paul II Hospital, Krakow, Poland; 2 Paediatric Heart Surgery Department and General Paediatric Surgery Department, Medical University of Warsaw, Warsaw, Poland; 3 Department of Radiology and Diagnostic Imaging, John Paul II Hospital, Krakow, Poland

**Keywords:** Tetralogy of Fallot, Long term follow up, Cardiac magnetic resonance, Echocardiography cardiopulmonary exercise test

## Abstract

**Background:**

Tetralogy of Fallot (ToF) is the most common cyanotic congenital heart disease and the population of ToF repair survivors is growing rapidly. Adults with repaired ToF develop late complications. The aim of this study was to describe and analyze long-term follow-up of patients with repaired ToF.

**Methods:**

This is a retrospective cohort study. Consecutive 83 patients with repaired ToF who did not undergo pulmonary valve replacement were included. Mean age of all patients was 30.5 ± 10.7. There were 49 (59%) male. Patients were divided into two groups according to the time since the repair (< 25 years and ≥ 25 years). The electrocardiographic (ECG), cardiopulmonary exercise testing (CPET), echocardiographic and cardiac magnetic resonance (CMR) data were reviewed retrospectively.

**Results:**

In CPET values were not significantly different in the two groups. In CMR volumes of left and right ventricles were not significantly different in the two groups. There were no differences between the groups in ventricular ejection fraction, mass of ventricles, or pulmonary regurgitation fraction. Among all the patients, ejection fraction and left and right ventricle mass, indexed pulmonary regurgitation volume measured by CMR did not correlate with the time since repair. In ECG among all the patients, ejection fraction of the RV, measured in CMR, negatively correlated with QRS duration (*r* = − 0.43; *p* < 0.001). There was a positive correlation between QRS duration and end diastolic volume of the RV (*r* = 0.30; *p* < 0.02), indexed end diastolic volume of the RV (*r* = 0.29; *p* = 0.04), RV mass (*r* = 0.36; *p* < 0.001) and left ventricle mass (*r* = 0.26; *p* = 0.04).

**Conclusion:**

Long-term survival and clinical condition after surgical correction of ToF in infancy is generally good and the late functional status in ToF – operated patients could be excellent up to 25 years after the repair. QRS duration could be an utility and easy factor to assessment of right ventricular function.

**Trial registration:**

The study protocol was approved by the local Ethics Committee. Each participant provided informed consent to participate in the study (license number 122.6120.88.2016 from 28.04.2016).

## Background

Tetralogy of Fallot (ToF) is the most common cyanotic congenital heart disease [1, 2]. Surgical correction of ToF has become the treatment of choice over four decades ago [3] and is well established [4]. As a result, survival has improved significantly and the population of ToF repair survivors is growing rapidly [1], with a 20–30-year survival rate at near 90% [1, 2, 5]. Nevertheless, survival at 30 years is lower than in the normal population [2], and the risk of death in the third postoperative decade is more than triple [5]. Adults with repaired ToF develop late complications, such as progressive exercise intolerance, arrhythmias, and heart failure [4, 6]. These complications are mainly due to pulmonary regurgitation, which leads to right ventricle dysfunction [7]. Previous studies have focused on outcomes of pulmonary valve replacement [8, 9] and risk factors assessment for adverse events [2, 10], but few have reported a long-term symptomatic status of patients after ToF repair.

The primary aim of this study was to describe and analyze long-term follow-up of patients with repaired ToF. The secondary aim was to assess the patients’ exercise capacity and type and frequency of complications.

## Methods

### Patient population

This is a retrospective cohort study. Consecutive patients with repaired ToF were included from the outpatients registry if they were > 18 years of age. Patients were referred for clinical assessment as part of routine clinical follow-up at the Department of Cardiac and Vascular Diseases, Institute of Cardiology Jagiellonian University College of Medicine, in the John Paul II Hospital, in Krakow. A main diagnosis was determined for every patient from hospital records, and patients were included in the study if they had only ToF as the primary diagnosis (ToF variants were excluded). To the further analysis 83 patients who did not undergo PVR were included. Patients were divided into two groups according to the time since the repair (< 25 years and ≥ 25 years). In patients population who did undergo PVR the data were obtained after PVR.

### Study protocol

All clinical and demographic variables were extracted from the patients’ medical records. Body mass index (BMI) was computed as weight/ height^2^ expressed in kg/m^2.^ [11]. Information on cardiac malformations, type of previous cardiac operations, age at surgical repair, reoperations, and current medications were recorded. The electrocardiographic (ECG), cardiopulmonary exercise testing (CPET), echocardiographic and cardiac magnetic resonance (CMR) data for the patients who did not undergo pulmonary valve replacement were reviewed retrospectively.

The study protocol was approved by the local Ethics Committee (license number 122.6120.88.2016). Each participant provided informed consent to participate in the study. All procedures were conducted in accordance with the ethical standards of the institutional and/or national research committee and with the 1964 Declaration of Helsinki and its later amendments, or comparable ethical standards. The study was funded by Jagiellonian Universtiy Collegium Medicum (CMUJ K/ZDS/007189; CMUJ K/ZDS/007820).

### ECG and Holter monitoring

Standard (25 mm/s speed and 1 mV/cm) 12-lead surface ECGs were analysed for rhythm, PQ interval and QRS duration (defined as the maximal QRS length in any lead from the first inflection to the final sharp vector crossing the isoelectric line). ECGs in patients with a pacemaker were excluded from this comparison. Twenty-four-hour Holter monitoring, performed with a Pathfinder SL version 1.7.1.4557, was analysed for rhythm and conduction abnormalities.

### Cardiopulmonary exercise testing

CPET was performed on a treadmill with a modified Bruce protocol (Reynolds Medical System, ZAN-600) as previously described [12]. To avoid pharmacologic influence, beta blockers were discontinued before CPET. Oxygen saturation and 12-lead ECG were continually monitored during the test, and blood pressure was measured manually every two minutes. Oxygen consumption (VO_2_) and carbon dioxide production (VCO_2_) were measured with computerized breath-by-breath analyzer. Peak oxygen uptake (VO_2_ peak) was defined as the highest value at peak workload, expressed in ml/kg/min and as % of predicted value.

### Echocardiography

During echocardiographic examination, ejection fraction of the left and right ventricle, according to the European Society of Cardiology guidelines, was measured [13]. Valves regurgitations and stenoses were evaluated with continuous wave Doppler and pulsed- wave Doppler. To estimate the severity of pulmonary stenosis, pulmonary gradient with continuous wave Doppler was measured. The severity of pulmonary and tricuspid regurgitation was estimated with the color Doppler. A quantitative assessment of the severity of pulmonary regurgitation was based on the deceleration velocity of the regurgitant flow, known as pressure half time (PHT). The PHT was measured with continuous wave Doppler. Function of the right heart was assessed by tricuspid annular-plane systolic excursion (TAPSE). Values of TAPSE lower than 18 mm were indicative of right ventricular longitudinal dysfunction [14]. Tissue Doppler imaging technique was applied at the tricuspid annulus to measure its systolic velocity (S′). The presence or absence of atrial and ventricle septal defect was checked. Images were obtained with Vivid 7 GE Medical System, USA.

### Cardiac magnetic resonance: imaging protocol

Breath-hold, ECG-gated imaging was performed by use of cardiac phased-array coil on 1.5 T whole-body scanner (Magnetom Sonata Maestro Class, Siemens, Erlangen, Germany) in left and right ventricle short-axis and axial views. After scout imaging was performed, cine biventricular imaging, using breath-hold steady-state free precision gradient echo technique, and flow-sensitive imaging at the pulmonary valve level, using free-breathing phase-contrast technique, were acquired. The imaging plane for a flow sequence was oriented perpendicularly to the main pulmonary artery at the pulmonary valve level. The velocity encoding was set at 100–550 cm/sec to avoid an aliasing artefact.

### Cardiac magnetic resonance: image analysis

Cine and flow images were assessed off-line with dedicated software package (MASS Medis, Leiden, the Netherlands). Left ventricular and right ventricular end-diastolic volume, end-systolic volume, ejection fraction, and myocardial mass were computed. End-diastolic volume, end-systolic volume and pulmonary regurgitation fraction were indexed to body surface area.

### Flow images

The forward flow and backward flow were calculated. Backward flow was considered to represent the volume of pulmonary regurgitation. Based on the forward and backward flow volumes, the fraction of pulmonary regurgitation (PRF) was calculated.

### Statistical analysis

Data were analyzed by use of statistical software package StatSoft STATISTICA 12.5. A *p* value of < 0.05 was considered statistically significant. Continuous variables are presented as mean ± SD or median with range. Student’s unpaired t-test and Mann-Whitney U test were used for comparison of continuous variables. All categorical variables were compared by use of the χ^2^ test. The age difference between the groups according to the time after the surgery was compensated for in statistical analysis. To assess the effects of the time-dependent covariates, repeated multivariate analyses of variance were performed. In case of significant results of these analyses, post-hoc testing was applied. Correlations between nominal variables were tested with Spearman’s rank correlation coefficient test or Pearson’s rank correlation coefficient test, depending on the distribution of interval variables.

## Results

### Population characteristics

The total cohort of ToF patients was 109. The median age of all patients was 28 years (interquartile range 19–64). There were 64 (59%) male. 57 (52.3%) of patients were in I NYHA class, 38 (34.9%) in II NYHA class and 14 (12.8%) in III NYHA class. The corrective surgery was performed between 1964 and 2012; the median patients age of intracardiac repair was 3.0 years (interquartile range 0.0–31.0 years). The mean time from repair to study was 26.1 ± 8.3 years. Twenty (18%) patients had received a palliative shunt (Blalock-Taussig repair) prior to complete repair. The most common surgical procedure was transannular patch, performed in 54 (49%) patients. Twenty-six patients were qualified for reoperation according to European Society of Cardiology guidelines [15] and underwent pulmonary valve replacement. The median age at PVR was 30.5 (interquartile range 19–53). To the further analysis 83 patients (76%), of median age of 26 years (interquartile range 19–64), who did not undergo PVR were included. None of the patients of this population had the criteria for reintervention at the time of the study. Patients were divided into two groups according to the time since the repair (< 25 years and ≥ 25 years). Table 1 illustrates the demographics and surgical characteristics of patients who did not undergo PVR after ToF repair.

**Table 1:** Baseline demographics and surgical characteristics of patients who did not undergo PVR

	All patients (*n* = 83)	Group 1 < 25 years since repair (*n* = 51)	Group 2 ≥ 25 years since repair (*n* = 32)	*P* values
Median age with interquartile range (yr)	26 (19–64)	25 (19–49)	37 (21–64)	< 0.001
Male/female	49/34	32/19	17/15	0.39
Height	168.3 ± 10.8	169.9 ± 12.2	165.8 ± 7.7	0.11
Weight	64.7 ± 14.8	63.5 ± 15.4	66.6 ± 14.0	0.40
BMI	22.8 ± 4.4	21.9 ± 4.5	24.1 ± 4.0	0.04
BSA	1.7 ± 0.2	1.7 ± 0.2	1.7 ± 0.2	0.94
Median age with interquartile range at ToF repair (yr)	3 (0–30)	3 (0–30)	3 (0–30)	0.66
Time since ToF repair (yr)	23 (6–53)	21 (6–24)	31 (25–53)	< 0.001
Number with prior Blalock – Taussig shunt	17	6	11	0.02
Number with transannular patch	39	29	10	0.02
NYHA functional class	NYHA I – 56	NYHA I – 38	NYHA I – 18	0.23
	NYHA II – 23	NYHA II – 13	NYHA II – 10	NYHA I – 0.08
	NYHA III – 4	NYHA III – 0	NYHA III – 4	NYHA II – 0.38
	NYHA IV – 0	NYHA IV –0	NYHA IV –0	NYHA III – 0.98

*BMI* Body mass index, *BSA* Body surface area, *NYHA* New York Heart Association

### Cardiopulmonary exercise testing

The results of cardiopulmonary exercise test are presented in Table 2. Patients who were ≥ 25 years since the repair had significantly lower peak heart rate. Other values were not significantly differe in the two groups.

**Table 2:** Results of patients’ cardiopulmonary exercise testing of patients who did not undergo PVR

CPET variable	Mean value ± SD (*n* = 83)	Group 1 < 25 years since repair (*n* = 43)	Group 2 ≥ 25 years since repair (*n* = 40)	*p* value
T [min]	16.3 ± 3.3.4	16.9 ± 3.6	15.5 ± 2.8	0.08
HR peak [beat/min]	157 ± 29	164 ± 29	146 ± 26	< 0.001
HR peak [%N]	70 ± 33	71 ± 29	69 ± 35	0.27
SBP peak [mmHg]	141 ± 23	141 ± 18	141 ± 30	0.96
DBP peak [mmHg]	73 ± 12	71 ± 12	75 ± 12	0.23
VO2 peak	1.8 ± 0.7	1.9 ± 0.7	1.7 ± 0.6	0.42
VO2 peak [%N]	69.1 ± 23.0	67.1 ± 23.5	72.0 ± 22.3	0.39
VO2/kg peak [ml/min*kg]	27.6 ± 8.2	28.6 ± 8.6	25.9 ± 7.5	0.18
VO2max/kg [%N]	76.3 ± 22.1	73.4 ± 22.3	80.5 ± 21.5	0.19
VCO2 peak	2.0 ± 0.8	2.0 ± 0.9	1.9 ± 0.7	0.34
VCO2 peak [%N]	57.5 ± 18.3	56.8 ± 19.5	58.4 ± 16.8	0.72
RER peak	1.1 ± 0.2	1.1 ± 0.2	1.1 ± 0.1	0.84

*T* Time, *HR* Heart rate, *SBP* Systolic blood pressure, *DBP* Diastolic blood pressure, *VO*_*2peak*_ Peak oxygen uptake, *VO*_*2peak*_*/kg* Peak oxygen uptake per kilogram, *VCO*_*2peak*_ Peak carbon dioxide uptake, *RER* Respiratory exchange ratio

### Echocardiography

Echocardiography was completed in all patients. The results of echocardiography are presented in Table 3.  Moderate or severe pulmonary regurgitation was present in 69 subjects (83%), and severe tricuspid regurgitation was present in 4 (5%). Hemodynamically insignificant residual ventricular septal defect, was present in 16 patients (19%).

**Table 3:** Results of patients’ echocardiography of patients who did not undergo PVR

Echocardiography parameter	Mean value ± SD (*n* = 83)	Group 1 < 25 years since repair (*n* = 51)	Group 2 ≥ 25 years since repair (*n* = 32)	*p* value
RVOT prox [mm]	30.5 ± 6.7	29.6 ± 5.8	31.9 ± 7.9	0.14
RDVs [mm]	36.1 ± 8.9	37.7 ± 9.3	33.9 ± 7.7	0.10
RDVd [mm]	71.6 ± 9.9	72.4 ± 10.6	70.3 ± 8.8	0.41
LVD [mm]	44.3 ± 5.9	43.9 ± 6.1	44.9 ± 5.6	0.45
LVS [mm]	28.2 ± 6.0	28.5 ± 4.7	27.7 ± 7.6	0.54
RA area [cm^2^]	19.3 ± 7.2	17.9 ± 4.9	21.5 ± 9.4	0.04
LA area[cm^2^]	14.9 ± 5.3	12.8 ± 2.3	17.8 ± 6.6	< 0.001
EF [%]	58.7 ± 20.3	57.9 ± 20.1	60.0 ± 20.8	0.64
PV gr.max [mmHg]	24.3 ± 21.2	22.5 ± 16.3	27.2 ± 27.1	0.33
PV gr.mean [mmHg]	14.0 ± 12.9	12.7 ± 8.6	16.0 ± 17.6	0.26
PV PHT	104.3 ± 58.9	91.1 ± 42.0	126.2 ± 75.2	0.01
Tricuspid annulus [mm]	36.5 ± 7.3	36.0 ± 7.6	37.4 ± 6.8	0.57
TAPSE [mm]	21.2 ± 4.3	21.3 ± 4.1	21.2 ± 4.5	0.94
S′ [cm/s]	11.7 ± 2.0	11.8 ± 1.9	11.4 ± 2.1	0.43
RVSP (mmHg)	38.8 ± 16.5	36.0 ± 12.3	42.2 ± 20.4	0.20

*RVOT* Right ventricle outflow track, *RDVs* Right ventricle dimension at end systole, *RDVd* Right ventricle dimension at end diastole, *LVD* Left ventricle diastolic dimension, *LVS* Left ventricle systolic dimension, *LA* Left atrium, *RA* Right atrium, *EF* Ejection fraction, *PV gr max* Maximal pulmonary valve gradient, *PV gr min* Minimal pulmonary valve gradient, *PV PHT* Pulmonary valve pressure half time, *TAPSE* Tricuspid annular plane systolic excursion, *S’* Systolic velocity of tricuspid annulus, *RVSP* Right ventricular systolic pressure

The patients with less than 25 years since the repair had significantly smaller right and left atria than did the patients operated before that time.

### Cardiac magnetic resonance

The results of cardiac magnetic resonance are presented in Table 4. Volumes of left and right ventricles were not significantly different in the two groups. Also, there were no differences between the groups in ventricular ejection fraction, mass of ventricles, or pulmonary regurgitation fraction.

**Table 4:** Results of patients’ cardiac magnetic resonance of patients who did not undergo PVR

CMR parameter	Mean value ± SD (*n* = 83)	Group 1 < 25 years since repair (*n* = 51)	Group 2 ≥ 25 years since repair (*n* = 32)	*p* value
LA area [cm^2^]	20.2 ± 5.1	19.6 ± 5.4	21.1 ± 4.5	0.18
RA area [cm^2^]	26.3 ± 8.2	24.9 ± 7.2	28.4 ± 9.2	0.06
RV area [cm^2^]	41.9 ± 10.3	43.2 ± 11.0	40.3 ± 9.4	0.42
LVD [cm]	4.8 ± 0.5	4.7 ± 0.5	4.8 ± 0.5	0.47
LVS [cm]	3.2 ± 0.5	3.2 ± 0.5	3.2 ± 0.5	0.72
RV EF [%]	50.7 ± 9.9	50.2 ± 8.9	51.5 ± 11.3	0.57
RV EDV [ml]	197.4 ± 61.9	202.6 ± 58.0	189.0 ± 67.8	0.33
RV EDV indexed [ml/m^2^]	112.9 ± 33.8	118.4 ± 28.4	103.8 ± 40.0	0.07
RV ESV [ml]	104.0 ± 47.8	95.4 ± 35.8	122. ± 65.5	0.17
RV ESV indexed [ml/m^2^]	59.8 ± 27.9	55.9 ± 16.8	66.7 ± 41.5	0.39
RV mass [g]	46.3 ± 19.5	47.3 ± 19.6	44.9 ± 19.5	0.59
RV mass indexed [g/m^2^]	27.5 ± 10.6	28.8 ± 10.6	25.7 ± 10.4	0.23
LV EF [%]	57.7 ± 7.6	56.6 ± 7.5	59.3 ± 7.4	0.11
LV EDV [ml]	129.8 ± 35.1	129.9 ± 36.9	129.6 ± 32.5	0.97
LV EDV indexed [ml/m^2^]	76.3 ± 17.0	76.3 ± 16.1	76.3 ± 18.7	0.99
LV ESV [ml]	56.1 ± 17.9	59.5 ± 18.9	49.7 ± 14.7	0.19
LV ESV indexed [ml/m^2^]	31.6 ± 8.2	33.6 ± 8.7	28.7 ± 6.6	0.19
LV mass [g]	100.9 ± 31.2	99.6 ± 33.4	102.8 ± 27.9	0.66
LV mass indexed [g/m^2^]	58.3 ± 14.8	58.3 ± 16.7	58.3 ± 11.8	0.98
PRV [ml]	54.4 ± 30.5	53.7 ± 26.8	55.6 ± 37.0	0.84
PRV indexed [ml/m^2^]	30.0 ± 17.2	28.0 ± 13.1	34.3 ± 23.7	0.30
PRF [%]	42.0 ± 10.3	42.6 ± 10.7	40.9 ± 10.1	0.67

*CMR* Cardiac magnetic resonance, *LA* Left atrium, *RA* Right atrium, *RV* Right ventricle, *LVD* Left ventricle diastolic dimension, *LVS* Left ventricle systolic dimension, *RVEDV* Right ventricle end-diastolic volume, *RVESV* Right ventricle end-systolic volume, *RVEF* Right ventricle ejection fraction, *RV mass* Right ventricle mass, *LVEF* Left ventricle ejection fraction, *LVEDV* Left ventricle end-diastolic volume, *LVESV* Left ventricle end-systolic volume, *LV mass* Left ventricle mass, *PRV* Pulmonary regurgitation volume, *PRF* Pulmonary regurgitation fraction

Among all the patients, ejection fraction and left and right ventricle mass, indexed pulmonary regurgitation volume measured by CMR did not correlate with the time since repair.

### ECG and Holter monitoring

**Fig 1:**
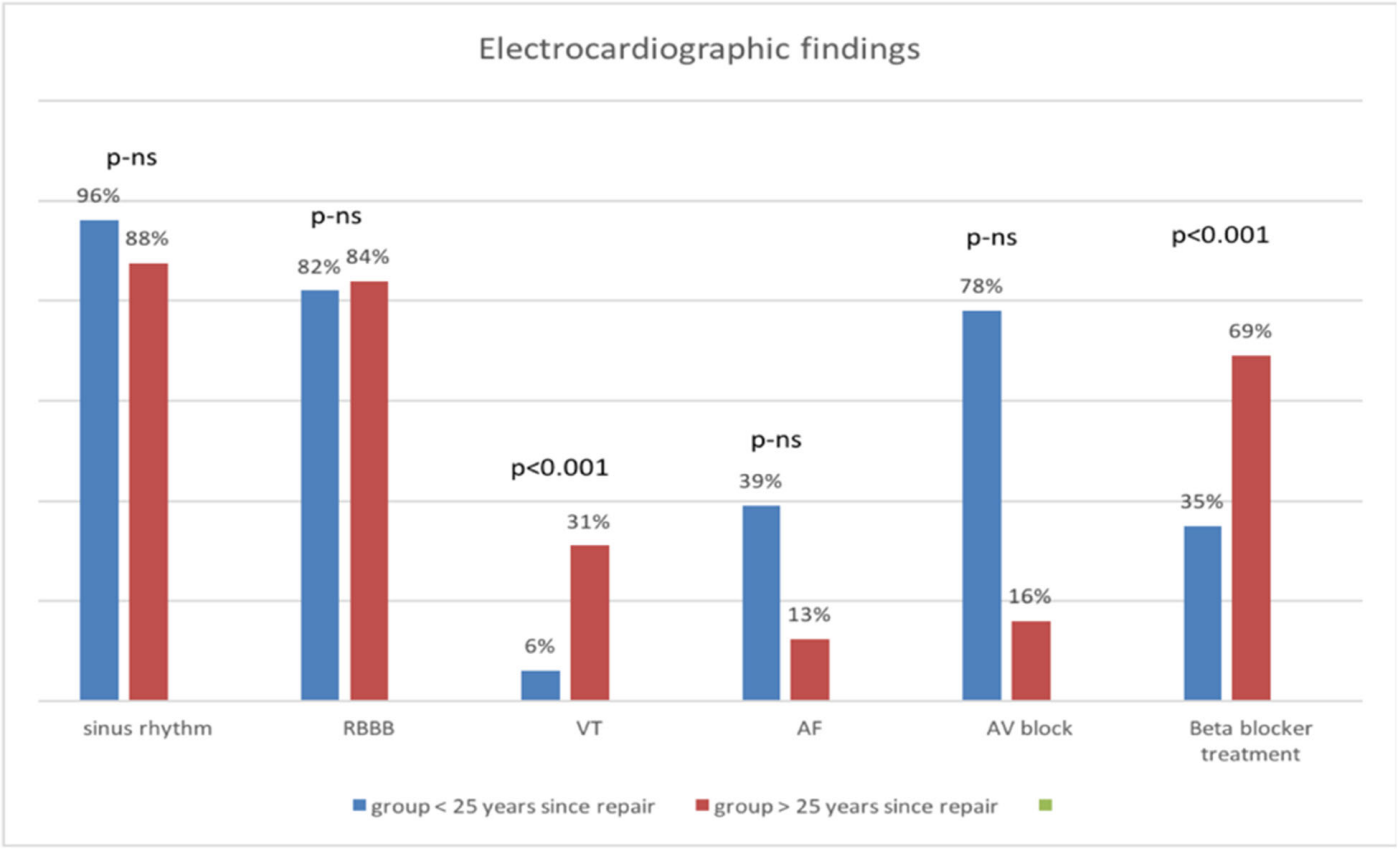
Patients’ ECG and Holter monitor findings. *AF* Atrial fibrillation, *AV* Atrioventricular, *RBB* Right bundle branch block, *VT* Ventricular tachycardia

Patients’ ECG and Holter monitor findings are illustrated in the figure below (Fig. 1). Among all the patients, 77 (93%) had sinus rhythm, 6 (7%) had persistent atrial fibrillation, which was the most common arrhythmia. Most of the patients (69, 83%) had right bundle branch block; 13 (17%) had episodes of ventricular tachycardia, 9 (11%) atrioventricular block; and 40 (48%) were treated with beta blocker.

Ventricular tachycardia and the need to use beta blocker medications were significantly less frequent in patients who were < 25 years since the repair as compared to operated later (13 vs 70; *p* = 0.002 and 40 vs 43; *p* = 0.003, respectively). There were no statistically significant differences between these groups in PQ interval duration (176 ms vs 191 ms; *p* = 0.3) or QRS interval duration (138 ms vs 132 ms; *p* = 0.5).

Among all the patients, ejection fraction of the right ventricle, measured in CMR, negatively correlated with QRS duration (*r* = − 0.43; *p* < 0.001). There was a positive correlation between QRS duration and end diastolic volume of the right ventricle (*r* = 0.30; *p* < 0.02), indexed end diastolic volume of the right ventricle (*r* = 0.29; *p* = 0.04), right ventricle mass (*r* = 0.36; *p* < 0.001) and left ventricle mass (*r* = 0.26; *p* = 0.04).

## Discussion

The main finding of the study is to show that the late functional status in ToF – operated patients could be excellent up to 25 years after the repair. However, after more than 25 years since the repair, significant changes can occur, manifesting itself as progressive RV dilation and dysfunction or clinical arrhythmia.

Good functional status up to 25 years after the operation corresponds with other reports of excellent long-term outcomes [17–19]. Most prior studies focused on risk factors for pulmonary valve regurgitation and preoperative or postoperative testing. In contrast with some studies [16], we did not evaluate patients who had undergone reoperation; thus, our data are unique in this regard.

In our cohort, exercise performance in both groups did not have significant differences in regard to time since repair. O’Meagher et al. [21] found that the age at ToF repair did not explain the disparity in exercise capacity. Others [20] have reported that patients after ToF repair have markedly depressed exercise capacity but in comparison with healthy controls.

Chronic right ventricular overload due to pulmonary valve insuficiency can lead to right ventricle dilatation, dysfunction of both ventricles and arrhythmias [19, 21–23]. Although previous studies documented that pulmonary regurgitation can be well tolerated for many years [24, 25], our data extend these observations and showed, as did Bacha et al. [23], that right ventricular volume can be preserved without deterioration for more than 25 years.

QRS duration is strongly associated with right ventricular function [10], as was first recognized by Gatzoulis et al. [26], and QRS prolongation has been correlated with the presence of ventricular arrhythmias [19]. In contrast to previous findings [10], we found that QRS interval was not correlated with the length of follow-up and was not prolonged after 25 years from date of operation, even though the presence of ventricular tachycardia increased with time. This should be obviously noted that beta blockers may affect the QRS. The relationship between QRS duration and right ventricular enlargement and mass was found primarily in studies of Book et al. [27] and Neffke et al. [28]. In our study, we confirmed a significant correlation between QRS duration and right ventricular mass and end diastolic volume in patients with ToF; also, we made the new finding of a strong negative relationship between QRS duration and right ventricular ejection fraction.

Supraventricular and ventricular tachycardia are well-documented complications of ToF repair [6, 24]. We found an increasing incidence of these arrhythmias with time from the repair. Abnormal right atrial size has been shown as a strong predictor of tachyarrhythmia [6], and we found significant increases in right atrial area with time since the repair.

Pulmonary regurgitation is a frequent late consequence of repair in ToF patients [25] is associated with right ventricle dilatation. In natural history, pulmonary regurgitation leads to ventricular dysfunction and heart failure developing over time [29]. Our study did not confirm time related impairment both ventricles ejection fractions and relationship to pulmonary regurgitation volume. This finding supports those of studies which showed that the right ventricle fails after 40 years of successful adaptation [19]. Moreover, in our study, in both groups right ventricle parameters were not adequate according to current guidelines to meet criteria of pulmonary valve replacement or right ventricular failure.

In conclusion, long-term survival and clinical condition after surgical correction of ToF in infancy is generally good. Our observations indicate an optimistic clinical function and exercise capacity in the patients operated more than 25 years ago. QRS duration could be an utility and easy factor to assessment of right ventricular function. Additionally longer prospective follow up is still needed and may provide a clearer understanding of this group.

### Study limitations

The limitations of the study should be mentioned. First, the small sample of patients population and retrospective cohort study. Second, this is a cross sectional study and the parameters and the history of adverse event was collected at the time of patient enrollment.

## Abbreviations

CMR: Cardiac magnetic resonance
CPET: Cardiopulmonary exercise testing
ECG: Electrocardiographic
PHT: Pressure half time
PVR: Pulmonary valve replacement
TAPSE: Tricuspid annular-plane systolic excursion
ToF: Tetralogy of Fallot
VO_2_ peak: Peak oxygen uptake

## References

[1] Geva T (2006). Indications and timing of pulmonary valve replacement after ToF repair. Semin Thorac Cardiovasc Surg Pediatr Card Surg Annu.

[2] Cuypers JAAE, Menting ME, Konings EEM, Opiü P, Utens EMWJ, Helbing WA (2014). The unnatural history of ToF: prospective follow-up of 40 years after surgical correction. Circulation.

[3] Gatzoulis MA, Balaji S, Webber SA, Siu SC, Hokanson JS, Poile C (2000). Risk factors for arrhythmia and sudden cardiac death late after repair of ToF: a multicentre study. Lancet.

[4] Oechslin EN, Harrison DA, Harris L, Downar E, Webb GD, Siu SS (1999). Reoperation in adults with repair of ToF: indications and outcomes. J Thorac Cardiovasc Surg.

[5] Geva T, Sandweiss BM, Gauvreau K, Lock JE, Powell AJ (2004). Factors associated with impaired clinical status in long-term survivors of ToF repair evaluated by magnetic resonance imaging. J Am Coll Cardiol.

[6] Dennis M, Moore B, Kotchetkova I, Lynne Pressley RC, DSC (2017). Adults with repaired tetralogy: low mortality but high morbidity up to middle age. Open Heart.

[7] Babu-Narayan SV, Diller G-P, Gheta RR, Bastin AJ, Karonis T, Li W (2014). Clinical outcomes of surgical pulmonary valve replacement after repair of ToF and potential prognostic value of preoperative cardiopulmonary exercise testing. Circulation.

[8] Therrien J, Siu SC, McLaughlin PR, Liu PP, Williams WG, Webb GD (2000). Pulmonary valve replacement in adults late after repair of ToF: are we operating too late?. J Am Coll Cardiol.

[9] Lee C, Kim YM, Lee C-H, Kwak JG, Park CS, Song JY (2012). Outcomes of pulmonary valve replacement in 170 patients with chronic pulmonary regurgitation after relief of right ventricular outflow tract obstruction. J Am Coll Cardiol.

[10] Scherptong RWC, Hazekamp MG, Mulder BJM, Wijers O, Swenne CA, Van Der Wall EE (2010). Follow-up after pulmonary valve replacement in adults with ToF: Association between QRS duration and outcome. J Am Coll Cardiol.

[11] Must A, Dallal GE, Dietz WH (1991). Reference data for obesity: 85th and 95th percentiles of body mass index (wt/ht2) and triceps skinfold thickness. Am J Clin Nutr.

[12] Tomkiewicz-Pajak L, Podolec P, Kostkiewicz M, Tracz W (2002). Lung function and exercise tolerance in patients with heart failure. Acta Cardiol.

[13] Lang RM, Badano LP, Mor-Avi V, Afilalo J, Armstrong A, Ernande L (2015). Recommendations for Cardiac Chamber Quantification by Echocardiography in Adults: An Update from the American Society of Echocardiography and the European Association of Cardiovascular Imaging. EHJ - Cardiovasc Imaging.

[14] O’Meagher Shamus, Seneviratne Martin, Skilton Michael R., Munoz Phillip A., Robinson Peter J., Malitz Nathan, Tanous David J., Celermajer David S., Puranik Rajesh (2015). Right Ventricular Mass is Associated with Exercise Capacity in Adults with Repaired Tetralogy of Fallot. Pediatric Cardiology.

[15] Michael A, Gatzoulis U, Gohlke C, Baerwolf GH, Kilner P (2010). ESC Guidelines for the management of grown-up congenital heart disease (new version 2010). The Task Force on the Management of Grown-up Congenital Heart Disease of the European Society of Cardiology (ESC). Eur Heart J.

[16] Cavalcanti PEF, Barros MP, Sá O, Cecília Y, Santos A (2013). Pulmonary valve replacement after operative repair of ToF meta-analysis and meta-regression of 3,118 patients from 48 studies. J Am Coll Cardiol.

[17] Katz N M, Blackstone E H, Kirklin J W, Pacifico A D, Bargeron L M (1982). Late survival and symptoms after repair of tetralogy of Fallot. Circulation.

[18] Murphy JG, Gersh BJ, Mair DD, Fuster V, McGoon MD, Ilstrup DM (1993). Long-term outcome in patients undergoing surgical repair of ToF. N Engl J Med.

[19] Nollert G, Fischlein T, Bouterwek S, Böhmer C, Klinner W, Reichart B (1997). Long-term survival in patients with repair of ToF: 36-year follow-up of 490 survivors of the first year after surgical repair. J Am Coll Cardiol.

[20] Dłużniewska N, Podolec P, Miszalski-Jamka T, Krupiński M, Banyś P, Urbańczyk M (2018). Effect of ventricular function and volumes on exercise capacity in adults with repaired ToF. Indian Heart J.

[21] O’Meagher S, Munoz PA, Alison JA, Young IH, Tanous DJ, Celermajer DS (2012). Exercise capacity and stroke volume are preserved late after tetralogy repair, despite severe right ventricular dilatation. Heart.

[22] Sabate Rotes Anna, Johnson Jonathan N., Burkhart Harold M., Eidem Benjamin W., Allison Thomas G., Driscoll David J. (2014). Cardiorespiratory Response to Exercise before and after Pulmonary Valve Replacement in Patients with Repaired Tetralogy of Fallot: A Retrospective Study and Systematic Review of the Literature. Congenital Heart Disease.

[23] Bacha EA, Scheule AM, Zurakowski D, Erickson LC, Hung J, Lang P (2001). Long-term results after early primary repair of ToF. J Thorac Cardiovasc Surg.

[24] Discigil B, Dearani JA, Puga FJ, Schaff HV, Hagler DJ, Warnes CA (2001). Late pulmonary valve replacement after repair of ToF. J Thorac Cardiovasc Surg.

[25] Hickey EJ, Veldtman G, Bradley TJ, Gengsakul A, Manlhiot C, Williams WG (2009). Late risk of outcomes for adults with repaired ToF from an inception cohort spanning four decades. Eur J Cardio-Thoracic Surg Oxford University Press.

[26] Gatzoulis MA, Till JA, Somerville J, Redington AN, Cordina R, Celermajer DS (1995). Mechanoelectrical interaction in ToF. QRS prolongation relates to right ventricular size and predicts malignant ventricular arrhythmias and sudden death. Circulation.

[27] Book WM, Parks WJ, Hopkins KL, Hurst JW (1999). Electrocardiographic predictors of right ventricular volume measured by magnetic resonance imaging late after total repair of ToF. Clin Cardiol.

[28] Neffke JGJ, Tulevski II, Van Der Wall EE, Wilde AAM, Van Veldhuisen DJ, Dodge-Khatami A (2002). ECG determinants in adult patients with chronic right ventricular pressure overload caused by congenital heart disease: relation with plasma neurohormones and MRI parameters. Heart.

[29] Bove T, Vandekerckhove K, Devos D, Panzer J, Groote K, De Wilde H (2014). Functional analysis of the anatomical right ventricular components: should assessment of right ventricular function after repair of ToF be refined?. Eur J Cardio-thoracic Surg.

